# Crosstalk between Zinc Status and *Giardia* Infection: A New Approach

**DOI:** 10.3390/nu7064438

**Published:** 2015-06-03

**Authors:** Humberto Astiazarán-García, Gemma Iñigo-Figueroa, Luis Quihui-Cota, Iván Anduro-Corona

**Affiliations:** 1 Departamento de Nutrición y Metabolismo, Coordinación de Nutrición, Centro de Investigación en Alimentación y Desarrollo, A.C. Carretera a La Victoria Km 0.6, Hermosillo, Sonora, CP 83304, Mexico; E-Mails: gemma@estudiantes.ciad.mx (G.I.-F.); ivan.anduro@ciad.mx (I.A.-C.); 2 Departamento de Nutrición Pública y Salud, Coordinación de Nutrición, Centro de Investigación en Alimentación y Desarrollo, A.C. Carretera a La Victoria Km 0.6, Hermosillo, Sonora, C.P. 83304, Mexico; E-Mail: lquihui@ciad.mx

**Keywords:** zinc supplementation, zinc deficiency, *Giardia lamblia*, giardiasis, parasite infection, micronutrient supplementation

## Abstract

Zinc supplementation has been shown to reduce the incidence and prevalence of diarrhea; however, its anti-diarrheal effect remains only partially understood. There is now growing evidence that zinc can have pathogen-specific protective effects. Giardiasis is a common yet neglected cause of acute-chronic diarrheal illness worldwide which causes disturbances in zinc metabolism of infected children, representing a risk factor for zinc deficiency. How zinc metabolism is compromised by *Giardia* is not well understood; zinc status could be altered by intestinal malabsorption, organ redistribution or host-pathogen competition. The potential metal-binding properties of *Giardia* suggest unusual ways that the parasite may interact with its host. Zinc supplementation was recently found to reduce the rate of diarrhea caused by *Giardia* in children and to upregulate humoral immune response in *Giardia*-infected mice; *in vitro* and *in vivo*, zinc-salts enhanced the activity of bacitracin in a zinc-dose-dependent way, and this was not due to zinc toxicity. These findings reflect biological effect of zinc that may impact significantly public health in endemic areas of infection. In this paper, we shall explore one direction of this complex interaction, discussing recent information regarding zinc status and its possible contribution to the outcome of the encounter between the host and *Giardia.*

## 1. Introduction

Nutrition plays a fundamental role in the maintenance of health and the treatment of disease. The ability of the immune system to prevent infection and disease is strongly influenced by the nutritional status of the host, and an inadequate intake of macronutrients or selected micronutrients may compromise its ability to resist infectious pathogens; however, nutrition does not influence all infections equally [1,2].

Nowadays, there is growing interest in dietary factors, in particular micronutrients, from the perspective of disease pathogenesis and potential for treatment. Results of field and laboratory studies provide convincing evidence that micronutrient deficiencies contribute to the mortality and morbidity of infectious diseases [1,2]. This awareness has led to the conduct of several micronutrient supplementation studies which have been successful in reducing illness and decreasing deaths. Micronutrients offer a potentially inexpensive feasible means of altering the outcome of infectious diseases in the developing world. The evidence is strongest for the essential mineral zinc, which has caught wide scientific attention for the conceptual promise it has to offer for prevention, control and treatment of childhood diarrhea.

Diarrhea is one of the most common health problems affecting children, it can lead to malnutrition, developmental disorders, and even death [3]. In 2004, the World Health Organization (WHO) and the United Nations International Children’s Emergency Fund (UNICEF) issued a global recommendation for the daily supplementation with 20 mg of zinc in children 6 months of age and older, and 10 mg of zinc in infants younger than 6 months for 10–14 days on diarrheal onset [3]. This recommendation was based on several randomized controlled trials, meta-analyses and reviews [4] that have demonstrated the utility of zinc supplementation to reduce the incidence and prevalence of diarrhea, as well as the severity of the current episode, prevent subsequent episodes and improve other diarrhea related outcomes. Nevertheless, the protective mechanism of zinc has remained elusive.

Zinc has been tested for its ability to treat and prevent diarrheal diseases in many large field trials over a period of over 4 decades and has generally been found effective. However, significant heterogeneity of zinc on diarrhea-related outcomes has been observed across trials [4,5,6]. Potential contributors to this heterogeneity are currently not fully understood. Patel *et al.* [7] conducted a systematic analysis of several large studies and showed that the influence of zinc supplementation in acute diarrhea differs by the isolated organism, and that the beneficial effect of zinc may not be equivalent against the common causative agents. At present, evidence has shown that zinc can have pathogen-specific protective effects [8,9,10,11]. These findings suggest that the current strategy of zinc supplementation may optimize the therapeutic benefit based on the causative organism, but further studies are required to support this. The interactions of nutrition and infection with regard to individual infections and defined nutrients are now better known. Giardiasis remains as a common yet neglected cause of acute and chronic diarrheal illness worldwide [12]. This infection has been related to disturbances in the zinc metabolism of infected children [13], and may represent a risk factor for zinc deficiency [14]. In this paper we shall explore one direction of this complex interaction, discussing recent information regarding zinc status and its possible contribution to the outcome of the encounter between the host and *Giardia*.

## 2. Zinc

### 2.1. Zinc Biology

Since its discovery as an important element for human health in the 1960s, zinc has been widely studied but many questions regarding its mechanism of action and utility still remain unanswered [15]. It is the second most abundant trace element in the human body and is required for its normal functioning [16]. It plays critical catalytic, structural, and regulatory roles [17]. Zinc enables hundreds of enzymes to function, facilitates protein synthesis and folding, and regulates processes such as gene expression and apoptosis [18]. Zinc is also important for DNA and RNA metabolism, as well as cellular replication, differentiation and growth [19]. Therefore, organs that are dependent on continuous cell division for proper function, such as the immune system and the gut, are particularly sensitive to zinc deficiency.

### 2.2. Zinc Deficiency

A regular intake of zinc is required as there are no large stores in the body from which it can be easily mobilized [20]. Zinc is widely distributed in foods. Meat, fish and poultry are the major contributors of zinc in the diet, although dairy products and cereals also contribute substantial amounts [21].

Zinc deficiency is largely related to inadequate intake or absorption of zinc from the diet. High levels of inhibitors in the diet, such as fibre and phytates (mainly found in plant-based diets), may result in low absorption of zinc, even though intake of zinc may be acceptable. In general, zinc absorption from a diet high in animal protein will be greater than from a diet rich in plant derived proteins [22].

Although severe zinc deficiency is rare nowadays, mild-to-moderate zinc deficiency is quite common throughout the world. It is estimated that 17% of the global population is at risk of inadequate zinc intake [23]. Regional estimated prevalence ranges from 7.5% to 30%, with specific countries in South and South-East Asia, Sub-Saharan Africa, and Central America having the greatest risk of inadequate zinc intake. Part of the problem is that many people do not eat enough zinc-rich foods, while the mineral is also not well absorbed.

Children are especially vulnerable to deficiency because their periods of rapid growth create increased zinc needs that may remain unmet [24]. Zinc is recognized as problem nutrient for the older breastfed infant because of the challenge of obtaining adequate intake from exclusive breastfeeding and the resultant dependence on complementary foods to meet dietary requirements [25,26]. Meat is an excellent source of zinc and increased use of animal products has been recognized as an option, but it has often been considered unrealistic [26,27]. The choices of complementary foods are affected by economic and socio-cultural factors like family dietary pattern, culture, customs, beliefs of food taboos, previous experience of feeding patterns, inadequate nutritional knowledge, among others [28,29]. The majority of culturally acceptable and affordable complementary foods are plant- and cereal-based with relatively high phytate content which decreases zinc bioavailability [29]. 

In the developing world there is not only a zinc shortage in food but intestinal parasites inhibit its absorption as well [30,31]. In resource-scarce settings, poor water and sanitation systems lead to frequent exposure to gastrointestinal pathogens and high rates of infectious disease and diarrhea [32,33]. For instance, diarrhea can compromise intestinal function and damage the gastrointestinal tract lining, thereby causing increased zinc intestinal excretion [34]. Furthermore, zinc deficiency has been shown to increase the susceptibility of infants and children to gastrointestinal infections due to its adverse effects on gastrointestinal tract structure and function [34]; as a result, a cycle of zinc deficiency and infection may develop.

### 2.3. Zinc and Immune Function

Nutritional importance of zinc has been known for a long time, but in the last decades its importance in immune modulation has arisen. Although its function as a structural component of many enzymes has been known for many years, current experimental evidence points to an additional function of the concentration of free or loosely bound zinc ions as an intracellular signal [35].

Zinc functions as a modulator of the immune response through its availability [36], which is tightly regulated by a network based on ZnT-ZIP proteins and metallothionein for storage [37]. When this mechanism is disturbed, zinc availability is reduced, altering survival, proliferation and differentiation of the cells of different organs and systems [36]. The immune system is one of the most highly prolifarating organs [38,39], and the activity of cells involved in both innate and adaptive immunity is modulated by zinc. These cells include monocytes, polymorphonuclear-, natural killer-, T-, and B-cells. T cell functions and the balance between the different T helper cell subsets are particularly susceptible to changes in zinc status [36,37].

While acute zinc deficiency causes a decrease in innate and adaptive immunity, chronic deficiency increases the production of inflammatory cytokines, influencing the outcome of a large number of inflammatory diseases [36]. In 2008, Prasad [40] reported that in both young adults and elderly subjects, zinc supplementation decreased oxidative stress markers and generation of inflammatory cytokines.

Lymphopenia and thymic atrophy, which were the early hallmarks of zinc deficiency in humans and higher animals, are now known to be due to high losses of precursor T and B cells in the bone marrow [41]. Changes in gene expression for cytokines, DNA repair enzymes, zinc transporters, signaling molecules, *etc.*, suggest that cells of the immune system are attempting to adapt to the stress of suboptimal zinc [42]. That these effects can be functionally significant is demonstrated by the increased susceptibility of zinc-deficient animals to a number of bacterial, viral, and parasitic challenges [43]; zinc-deficient persons also experience increased susceptibility to a variety of pathogens [40].

Impaired immune functions due to zinc deficiency are shown to be reversed by an adequate zinc supplementation, which must be adapted to the actual requirement. Care must be taken on this matter as high dosages of zinc evoke negative effects on immune cells and show alterations that are similar to those observed with zinc deficiency [39].

It is clear that zinc affects multiple aspects of the immune system, from the barrier of the skin to gene regulation within lymphocytes [43]. Better understanding of the molecular and cellular changes made in response to inadequate zinc should lead to the development of immunotherapeutic interventions [42].

### 2.4. Zinc and Intestinal Parasitic Infections

Micronutrient deficiencies and parasitic diseases have similar geographical distribution with the same people experiencing both health problems in their lives. Intestinal parasitic infections are especially problematic because they have negative lifelong health consequences [44]; these infections can contribute to malnutrition which in turn results in delayed physical development and cognitive growth. Infection generally causes sequestration of zinc in the liver, and conditions that affect intestinal function and integrity can influence zinc homeostasis [21]. Parasitic infections are thought to contribute to child malnutrition through subtle reduction in digestion and absorption, chronic inflammation and loss of nutrients. Parasites may affect the intake of food, its subsequent digestion and absorption, metabolism and the maintenance of nutrient pools [30]. The most important parasites related to nutritional status are soil transmitted helminthes,protozoa such as *Giardia lamblia* and *Entamoeba histolytica*, followed by other parasites such as coccidia, *Schistosoma* sp. and malarial parasites [30]. Management guidelines for treatment of undernutrition in children explicitly recognize that treatment of overt and occult infection is a first step in breaking the cycle of infection, undernutrition, and immune impairment [45].

## 3. Giardiasis and Zinc Status

### 3.1. Giardia Lamblia Infection

Giardiasis is a major protozoan infection associated to diarrheal disease worldwide. The flagellate protozoan *Giardia lamblia* (*G. lamblia*) (synonym *Giardia intestinalis*, *Giardia duodenalis*), its causative agent, is the most commonly identified intestinal parasite in the United States and the most common protozoal intestinal parasite isolated worldwide [46,47,48,49]. In developing countries, the prevalence of giardiasis commonly ranges from 20% to 30%, with reports of 100% prevalence in some populations [50]. In some regions of the world, giardiasis is endemic and infection is practically universal by 2 years of age [51].

*Giardia* is transmitted through the ingestion of cysts in contaminated food or water, or directly via the fecal/oral route [51]. A striking feature of giardiasis is the spectrum of clinical symptoms that occur in infected individuals, from asymptomatic, to acute or chronic diarrheal disease associated with intestinal malabsorption, abdominal pain and nausea [12]. Multiple factors have been proposed to account for the disease variability, including the state of the host immune system, host age and nutritional status, strain genotype, infectious dose and, possibly co-infections [52,53,54].

Immune responses to *Giardia* occur in the intestinal mucosa and a spectrum of inflammatory mechanisms company this infection [12,52]. Secretory antibodies of the IgA class are important candidate for immune defense against *Giardia*, because they are secreted in large quantities into the intestinal lumen and their actions are antigen-specific [53]. 

In recent years, this protozoan attracted and renewed scientific attention because of the recognition of pathologies beyond the regular symptoms of infection––reviewed by Halliez and Buret [50]. These included chronic fatigue [55], post-infectious irritable bowel syndrome [55], and particularly, in early childhood, poor cognitive function and failure to thrive [56]. In addition, the inclusion of *Giardia* in the WHO’s Neglected Diseases Initiative in 2004 [57] and its re-emergence in industrialized countries, because of its recognized role in numerous outbreaks of diarrheal disease in day-care centers and in waterborne infections [58], led to a greater appreciation of the public health consequences of giardiasis.

Despite significant advances in the knowledge of the biochemistry and molecular biology of *Giardia*, little is known about its pathogenesis, where a combination of parasitic factors and host responses seems to be involved [59]. The pathophysiological consequences of *Giardia* infection include heightened rate of enterocytes apoptosis, shortening of brush border microvilli with villous atrophy, disaccharidase deficiencies, intestinal barrier dysfunction, activation of host lymphocytes, small intestinal malabsorption, anion hypersecretion and increased intestinal transit rates [12,60,61,62,63,64,65,66,67]. All these consequences are clearly multifactorial, and involve both host and parasite factors, as well as immunological and non-immunological mucosal processes [50].

### 3.2. Giardiasis and Zinc Deficiency

The interactions of nutrition and infection with regard to individual infections and defined nutrients are now better known. The association between zinc deficiency and giardiasis has scarcely been investigated although the association of giardiasis with undernutrition and malabsorption of micronutrients such as vitamin A [68,69,70] is well recognized.

Zinc is an element which cannot be stored in the body and therefore it can easily decline during the course of infective diseases [71]. In 1993 giardiasis was reported as a first-time risk factor for zinc malabsorption in children [13]. According to Jendryzcko *et al.* [13] disturbances were found in the zinc metabolism of *Giardia*-infected children. Elimination of zinc via urine, and serum-erythrocyte-zinc concentration were lower in infected children compared to non-infected; concentration of serum zinc carriers, total protein, albumin fraction and transferrin were not differing between both studied groups. Authors concluded that children with giardiasis had lower zinc absorption from the gastrointestinal tract, which led to zinc deficiency.

Several studies conducted regarding trace elements in giardiasis have also shown a significant decrease in zinc levels. Yones *et al.* [72] studied the effect of enteric parasitic infections on serum trace elements and nutritional status in Egyptian children; stunting, wasting and coincident decrease in serum zinc levels were more prominent among *Giardia lamblia* patients. Another study in Egyptian children [73] also reports affection of weight, intermittent diarrhea and significantly decreased serum zinc levels in the infected group compared to control. Two studies from Turkey [74,75] showed that serum zinc levels were significantly lower among the children, 2 years to 14 years old, with chronic giardiasis compared to their matched *Giardia*-free group. Zarebavani *et al.* [76] compared mean serum levels of immunological and biochemical parameters between *Giardia*-positive and *Giardia*-negative Iranian children, founding that zinc level in *Giardia* patients was remarkably lower, with no significant difference in serum levels of vitamin B12 and folic acid. In a recent study by Lazarte *et al.* [31], trace elements status was evaluated and associated to the presence of intestinal parasites in a group of children from a rural area of Bolivia. A multiple regression model showed the significant effect of the presence of the parasite *Giardia lamblia* on the serum zinc levels.

Quihui *et al.* [14] investigated the association between giardiasis and zinc deficiency in schoolchildren from northwestern Mexico. Longitudinal analysis demonstrated a significant increase of the serum zinc levels in the *Giardia*-infected group six months after treatment, even though no difference was observed in the socioeconomic characteristics and daily intakes of zinc between the groups. Another study from Turkey [77] found a significant increase in the serum zinc levels after treatment in 20 *Giardia*-infected children, 3 months to 14 years old. 

How zinc metabolism is compromised by *Giardia* is not well known. During infection the mucosal epithelium has a high turnover rate and functional immaturity of enzyme and transport systems. Thus, it is hypothesized that the increased intestinal absorption of zinc associated with anti-*Giardia* treatment may be explained by the restoration of the impaired intestinal mucosa as a result of the infection [78]. Another hypothesis has suggested that zinc deficiency may result from organ redistribution of zinc, from plasma to the liver, as part of the acute phase response of the host; apparently, the immune response of the host leads to activation of the synthesis of metallotionein in the liver and other tissues, altering the hepatic uptake of zinc [79,80].

Competition between the host and the pathogen for an important nutritional resource may be another way by which *Giardia* alters zinc status; in this situation the parasite and the host make great efforts to control zinc’s availability. Recent studies have shed some light on the role of zinc in gut infections. In order to survive within a host, some gut pathogens may evolve specialized systems to gain an advantage. Such is the case of *Salmonella* *thyphimurium*, a pathogen that thrives in the inflamed intestine by overcoming calprotectin-mediated zinc chelation through the expression of a high affinity zinc transporter. These findings highlight the importance of zinc acquisition in bacterial intestinal colonization [81].

The surface and flagella of the *Giardia* trophozoite are covered by a protein coat composed of a single variant-specific surface protein (VSP) [82,83]; these are metal-binding proteins and different cloned trophozoite lines all bound zinc [84]. The benefit of metal binding of VSPs to *Giardia* is unknown. Metal ions could maintain structure, bind neighboring VSPs or prevent oxidation; most interestingly, no other surface-residing Zn-finger protein exists in any other organism [85].

This group of proteins forms the major component of interface between *Giardia* and its host. VSPs can vary spontaneously or in response to antibody [86] or environmental selection [87], and loss of a VSP leads to replacement with another, which is usually immunologically distinct [86,88]. *Giardia* undergoes antigenic variation as a mechanism assumed to allow parasites to evade the host’s immune response, producing chronic and/or recurrent infections [84]. Because *Giardia’s* mechanism of protection may depend on switching expression among immunologically distinct VSPs, the host should be able to prevent infection by simultaneously developing specific immune responses to all variable surface molecules [85]. Despite the frequency of VSP switching, certain features are common to all known VSPs. An interesting idea at this point would be how the zinc status of the host could affect the mechanism controlling VSP switching during *Giardia* infection*,* and eventually its survival within the host.

As discussed by Nash *et al.* [84], in some infections the parasite burden is large, and the villi of the small intestine are covered with trophozoites. VSPs could bind zinc and inhibit the function of zinc or metal-requiring intestinal enzymes, or compete with the host for zinc and contribute to zinc malnutrition, which is increasingly recognized and occurs in populations and areas where *Giardia* is prevalent. In such scenario, only the presence of enough trophozoites, but not necessarily the presence of disease, would be needed to cause zinc malnutrition. The potential metal-binding properties of *G. lamblia* suggest unusual ways that the parasite may interact with its host, but whether this sequestration could affect the patient is not clear, especially in view of the non-specificity of metal binding by the VSPs [89].

### 3.3. Zinc Treatment and Giardiasis

Persistence of intestinal parasitic infections during de-worming campaigns in schoolchildren has been reported [90]. Rapid reinfection by *Giardia lamblia* after treatment is a common problem in endemic areas [91]. The front line treatment for giardiasis is antimicrobial therapy. Although the infection is usually self-limiting and recovery occurs in the majority of cases, there is a need for safe and effective ways of preventing and treating this disease. Several classes of antimicrobial drugs are available for the treatment of giardiasis. Among the most commonly used are members of the nitroimidazole family such as metronidazole and tinidazole [92]. However, first-line therapy fails in up to 20% of cases and cross-resistance between different agents can occur [93,94,95]. Alternative agents exist for treatment failures and for special circumstances (e.g., pregnancy), but these are generally less effective than nitroimidazole drugs [96].

Therefore, because of the prevalence of giardiasis and limited treatment options new interventions are required. Andrews and Mylvaganam [97] tested the sensitivity of *Giardia lamblia* to bacitracin and its zinc salt *in vitro*. The activity of bacitracin was enhanced 5–10 times by equimolar concentrations of zinc. This enhancement was not due to zinc toxicity and was zinc dose dependent. Significant *in vivo* activity was also demonstrated in a clinical trial in patients infected with the protozoa [98]. Nash and Rice [88] showed efficacy of zinc-finger-active drugs against *Giardia lamblia*. Zinc-finger-active compounds at 300 µM or less inhibited *Giardia lamblia* growth. In the adult mouse model, significant *in vivo* activity was demonstrated by increased cure rates and decreased parasite burdens. Because of high reinfection rate after traditional treatment and lack of effect on nutritional status or growth, anti-*Giardia* drug treatment of asymptomatic carriers generally is not recommended. 

Zinc supplementation was recently found to reduce the rate of diarrhea caused by giardiasis [11,99]. A randomized, double-blind, placebo-controlled trial [11] was conducted among Mexican children who were assigned to receive either vitamin A, a daily zinc supplement, a combined vitamin A and zinc supplement, or a placebo; then children were followed for 1 year. *G. lamblia* infections were reduced among children in the combined vitamin A and zinc group or the zinc alone group. In a study by Veenemans [99], there was no evidence that the efficacy of zinc supplements in reducing diarrhea rates is enhanced by concurrent supplementation with other micronutrients. Two other trials that investigated the added benefit of multinutrients in addition to zinc in preventing episodes of diarrhea also failed to show an advantage of combined supplementation above supplementation with zinc alone [100,101,102]. Therapeutic application of zinc as a pharmacological agent during infection seems to be an interesting idea, but all the different impacts of zinc treatment on the host must be considered in order to achieve a desired therapeutic effect [103].

Our group work using an experimental murine model of giardiasis [104] showed that *Giardia*-infected mice fed zinc-low or zinc-adequate diets had a significant growth retardation in comparison to non-infected controls. Supplementation of the diet with high levels of zinc improved growth performance, by increasing the body weight gain, and up-regulated the host’s humoral immune response by improving specific antibodies production. Clinical outcomes of zinc supplementation during giardiasis included significant weight gain, earlier and higher *Giardia*-specific antibody response and improved serum zinc levels despite the ongoing infection. These findings probably reflect biological effect of zinc that could be of public health importance in endemic areas of infection. Therefore, safe and inexpensive measures such as supplementation with oral zinc are apparently an attractive management and treatment option.

## 4. Conclusions

Zinc supplementation has been shown to reduce the incidence and prevalence of diarrhea. There is growing evidence indicating that zinc can have pathogen-specific protective effects. There is a need for additional research to understand the effect of zinc supplementation on diarrhea associated to different pathogens in order to use zinc treatment depending on the relative prevalence of causative organisms among a specific population. Intestinal parasites remain an important worldwide public health problem. *Giardia lamblia* is one of the important intestinal parasites associated to acute and chronic diarrheal diseases in human. The nutritional problems associated with persistent diarrhea seem to be more severe and less easily managed than those accompanying acute diarrhea. Giardiasis appears to be an interesting disease model to study the mechanisms by which zinc deficiency might contribute to pathogenesis of diarrhea or the success achieved in diarrhea control and treatment by zinc supplementation. Further studies are in progress to confirm the benefit of zinc supplementation during the acute phase of the disease. Understanding the mechanisms by which zinc contributes to the outcome of the encounter between an individual and an infectious agent requires additional research. Such understanding could bring micronutrient research from broad supplementation programs to potentially targeted nutritional therapy in specific infectious diseases.
